# Draft genome sequence of a multidrug-resistant *Enterobacter cloacae* strain isolated from a dog in Bangladesh

**DOI:** 10.1128/mra.01013-26

**Published:** 2026-10-01

**Authors:** Mukta Das Gupta, Ashutosh Das, Mishuk Shaha, Keya Ghosh, Snigdha Das

**Affiliations:** 1 Department of Microbiology and Veterinary Public Health, Faculty of Veterinary Medicine, Chattogram Veterinary and Animal Sciences University 130057, Chattogram, Bangladesh; 2 Genetics, Genomics and Health Research Group, Chattogram Veterinary and Animal Sciences University 130057, Chattogram, Bangladesh; 3 Department of Genetics and Animal Breeding, Faculty of Veterinary Medicine, Chattogram Veterinary and Animal Sciences University 130057, Chattogram, Bangladesh; Queens College Department of Biology, Queens, New York, USA

**Keywords:** *Enterobacter cloacae*, antibiotic resistance, zoonotic risk, dog, whole-genome sequencing

## Abstract

We report the draft genome sequence of a multidrug-resistant *Enterobacter cloacae* strain (3251) isolated from a dog in Bangladesh. The 5.1-Mb genome contains 41 predicted antimicrobial resistance genes and 9 virulence genes, highlighting companion animals as potential reservoirs of clinically relevant antimicrobial resistance.

## ANNOUNCEMENT

*Enterobacter cloacae* is an opportunistic pathogen responsible for a growing number of healthcare- and community-associated infections and is increasingly recognized for its multidrug-resistant (MDR) phenotype (1, 2). Companion animals, including dogs, can act as reservoirs of MDR *E. cloacae* and contribute to the dissemination of antimicrobial resistance genes through close human-animal contact (3, 4). Here, we report the draft genome of an MDR *E. cloacae* strain isolated from a dog in Bangladesh, providing insight into the resistome of a canine-associated isolate and its potential zoonotic relevance.

Between August and December 2023, a cloacal swab was collected from a dog in Chattogram District, Bangladesh, enriched in buffered peptone water (37°C, 24 h), and cultured on MacConkey agar with cefotaxime (2 µg/mL); a lactose-fermenting colony was selected for further characterization. The colony was subcultured onto blood agar (Columbia agar base with 5% sheep blood) and incubated for 18 h at 37°C, and genomic DNA was extracted from a well-isolated single colony directly from this subculture using the FavorPrep Tissue Genomic DNA Extraction Kit (Favorgen Biotech, Taiwan). A library was prepared with the Nextera XT DNA Library Preparation Kit (Illumina) and sequenced on an Illumina NextSeq 2000 platform (2 × 150 bp paired-end reads).

Reads were quality-filtered with Trimmomatic v0.39 (sliding window of 4 bp, minimum average Phred score of 20) (5) and assembled *de novo* with Unicycler v0.5.1 (6). Assembly quality was assessed with QUAST v5.2.0 (7), and the genome was annotated with the National Center for Biotechnology Information Prokaryotic Genome Annotation Pipeline (PGAP) v6.8 (8); default parameters were used unless stated otherwise. The isolate shared 98.86% average nucleotide identity with *E. cloacae* GCA_000755525.1 (JSpeciesWS, web server) (9) and a digital DNA-DNA hybridization value of 89.7% (GGDC formula d0, TYGS) (10), identifying it as *E. cloacae* ATCC 13047. Sequence type was determined with MLSTFinder v1.0.1 (11), pathogenicity potential with PathogenFinder v1.1 (12), plasmid content with PlasmidFinder v2.0.1 (13), mobile genetic elements with mobileOG-db v1.6 (14), prophages with PHASTEST v3.0 (15), antimicrobial resistance genes with CARD v3.2.9 (16) and ResFinder v4.7.2 (17), virulence genes with VirulenceFinder v3.2.1 (18), siderophores with antiSMASH v7 (19), CRISPR arrays and Cas proteins with CRISPRCasFinder v4.2.20 (20), and metabolic features with RAST v2.0 (21); genome visualization used Proksee (web server) (22).

**Fig 1:**
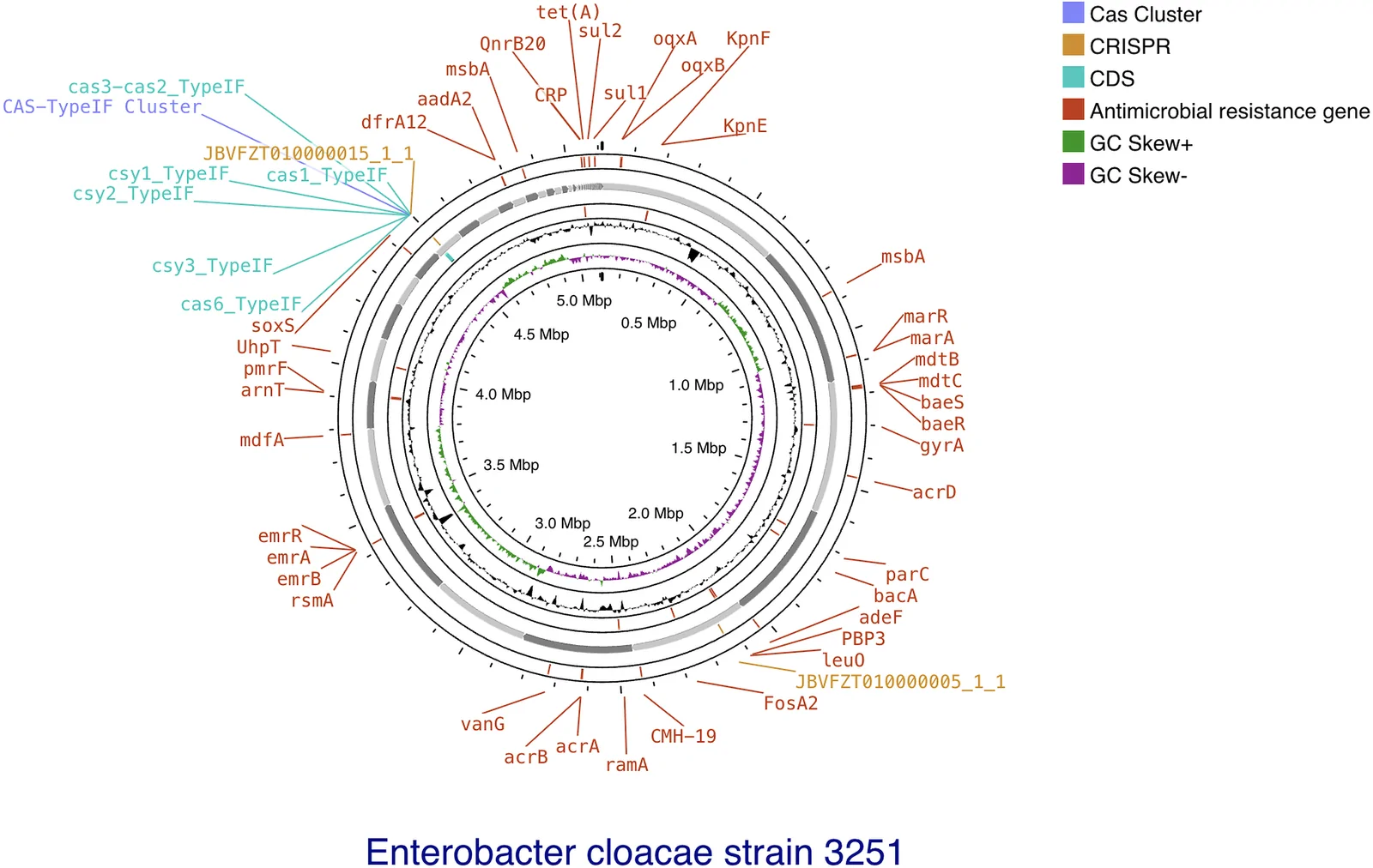
Circular representation of the draft genome assembly of *Enterobacter cloacae* strain 3251, showing predicted antimicrobial resistance genes and the CRISPR-Cas system (visualized with Proksee).

The 5.1-Mb draft genome (100 contigs, N50 = 388.2 kb, 54.5% GC content, 102× coverage) contained 4,969 predicted genes, 41 antimicrobial resistance genes conferring predicted resistance to 10 antibiotic classes, 9 virulence factor genes, 1 plasmid, 6 prophage regions, and 2 CRISPR arrays with 6 Cas proteins (Table 1). Illustrated genomic features are presented in Fig. 1. These findings expand the genomic resources available for canine-associated *E. cloacae* and support continued One Health surveillance of antimicrobial resistance at the human-animal interface.

**TABLE 1:** Genome properties of the multidrug-resistant *Enterobacter cloacae* strain 3251 isolated from a dog in Bangladesh

Genome feature	Value
Strain	*Enterobacter cloacae strain 3251*
BioSample accession no.	SAMN55422049
GenBank accession no.	JBVFZT000000000
Total no. of reads	3,458,782
Coverage (×)	102
Genome size (bp)	5.1 Mb
No. of contigs	100
Completeness of genome assembly	98.95%
Multilocus sequence type	3251
N50	388.2 kb
GC content (%)	54.5
Genes (total), PGAP	4,969
Pseudogenes (total)	51
Coding Sequence (CDS) (total)	4,878
CDS with protein	4,827
CDS without protein	51
Ribosomal RNA (5S, 16S, 23S)	2, 2, 6
Transfer RNA	73
Non-coding RNAs	7
No. of RNAs	90
No. of RAST-predicted subsystems	385
Predicted virulence genes	9
No. of prophages	6 (3 intact, 2 incomplete, 1 questionable)
No. of CRISPR arrays	2
No. of Cas proteins	6
Mobile genetic elements	420
Plasmids	1
Predicted antimicrobial resistance genes (CARD)	41
Predicted resistance phenotypes	Aminoglycosides, β-lactams, fosfomycin, fluoroquinolones, trimethoprim, chloramphenicol, sulfonamides, tetracyclines, polymyxins, macrolides
Predicted siderophores	Non-ribosomal peptide (NRP)-metallophores, non-ribosomal peptide synthetases (NRPS) and NRPS-independent siderophores, IucA/IucC-like siderophores, (thio)azol(in)e-containing peptides, linear and macrocyclic butyrolactones, terpene precursors, aryl polyenes

## Data Availability

This Whole Genome Shotgun project has been deposited in GenBank under the accession no. JBVFZT000000000. The version described in this paper is the first version, JBVFZT010000000. The associated BioProject accession number is PRJNA1425508, BioSample accession number is SAMN55422049, and the SRA accession number is SRP729396.
